# Mechanisms affecting the implementation of a national antimicrobial stewardship programme; multi-professional perspectives explained using normalisation process theory

**DOI:** 10.1186/s13756-020-00767-w

**Published:** 2020-07-02

**Authors:** Kay Currie, Rebecca Laidlaw, Valerie Ness, Lucyna Gozdzielewska, William Malcom, Jacqueline Sneddon, Ronald Andrew Seaton, Paul Flowers

**Affiliations:** 1 Glasgow Caledonian University, Glasgow, UK; 2 Health Protection Scotland, Glasgow, UK; 3 Healthcare Improvement Scotland, Glasgow, UK; 4 Queen Elizabeth University Hospital, NHS Greater Glasgow & Clyde, Glasgow, UK

**Keywords:** Antimicrobial stewardship programme, Multi-professional perspectives, Qualitative research, Normalisation process theory

## Abstract

**Background:**

Antimicrobial stewardship (AMS) describes activities concerned with safe-guarding antibiotics for the future, reducing drivers for the major global public health threat of antimicrobial resistance (AMR), whereby antibiotics are less effective in preventing and treating infections. Appropriate antibiotic prescribing is central to AMS. Whilst previous studies have explored the effectiveness of specific AMS interventions, largely from uni-professional perspectives, our literature search could not find any existing evidence evaluating the processes of implementing an integrated national AMS programme from multi-professional perspectives.

**Methods:**

This study sought to explain mechanisms affecting the implementation of a national antimicrobial stewardship programme, from multi-professional perspectives. Data collection involved in-depth qualitative telephone interviews with 27 implementation lead clinicians from 14/15 Scottish Health Boards and 15 focus groups with doctors, nurses and clinical pharmacists (n = 72) from five Health Boards, purposively selected for reported prescribing variation. Data was first thematically analysed, barriers and enablers were then categorised, and Normalisation Process Theory (NPT) was used as an interpretive lens to explain mechanisms affecting the implementation process. Analysis addressed the NPT questions ‘*which group of actors have which problems*, *in which domains, and what sort of problems impact on the normalisation of AMS into everyday hospital practice’*.

**Results:**

Results indicated that major barriers relate to organisational context and resource availability. AMS had coherence for implementation leads and prescribing doctors; less so for consultants and nurses who may not access training. Conflicting priorities made obtaining buy-in from some consultants difficult; limited role perceptions meant few nurses or clinical pharmacists engaged with AMS. Collective individual and team action to implement AMS could be constrained by lack of medical continuity and hierarchical relationships. Reflexive monitoring based on audit results was limited by the capacity of AMS Leads to provide direct feedback to practitioners.

**Conclusions:**

This study provides original evidence of barriers and enablers to the implementation of a national AMS programme, from multi-professional, multi-organisational perspectives. The use of a robust theoretical framework (NPT) added methodological rigour to the findings. Our results are of international significance to healthcare policy makers and practitioners seeking to strengthen the sustainable implementation of hospital AMS programmes in comparable contexts.

## Contributions to the literature

- Many studies have explored influences on individuals’ antibiotic prescribing behaviour or evaluated the effectiveness of specific interventions intended to improve prescribing; the impact of implementation efforts on the sustainability of interventions in an organisational context is not well known.
- This study looked beyond measured effectiveness of specific interventions, towards understanding the mechanisms by which these interventions can be accepted, adopted, and routinely embedded in clinical practice.
- Our findings contribute to filling a gap in the evidence by explaining the factors that act as barriers or enablers to the processes of implementing and sustaining improved prescribing in a complex, multi-professional setting.

## Background

The growing resistance of an increasing number of organisms to antimicrobial drugs used to prevent or treat infection has been characterised as a major threat to public health and “a threat to global stability and national security” [1](p.1), challenging the future delivery of modern medicine as we know it [2–4]. A variety of drivers to antimicrobial resistance (AMR) have been proposed, with inappropriate use of antibiotics recognised as a significant factor [1, 5]. Antimicrobial stewardship (AMS) can be defined as “making the best use of antimicrobials to manage infection so as to ensure optimal outcomes and minimal harm to patients and the wider society” [6] or “a coherent set of actions which promote using antimicrobials responsibly” [7](p.793). AMS is a key component of global strategies to combat AMR [1, 8]. These global strategies have been translated into local interventions via national AMS programmes [9, 10]. In Scotland, this led to the introduction of an Antimicrobial Management Team (AMT) in each regional Health Board, led by a medical infection specialist and an AMS pharmacist [11]. The work of the AMTs is co-ordinated nationally by the Scottish Antimicrobial Prescribing Group (SAPG), who collaborate with local teams to develop, disseminate and monitor the impact of strategies and guidelines to improve prescribing quality. The AMS programme in this study was established to deliver the first Scottish Action Plan in 2008 [11] and our evaluation follows ten years of evolving implementation.

The effectiveness of AMS interventions in changing prescribing behaviour and impacting on associated clinical outcomes has been reported in several studies. A recent systematic review [12] highlighted the potential impact of in-patient AMS programmes in significantly reducing the incidence of a range of antibiotic resistant organisms, an important clinical outcome. An updated Cochrane review [13], reported that targeted interventions to improve antibiotic prescribing to hospital in-patients are effective in increasing compliance with antibiotic policy and reducing duration of antibiotic treatment, thereby influencing key drivers to AMR. The review also concluded that future research should be directed towards exploring the barriers and enablers to the implementation of interventions. Furthermore, the sustainability of AMS interventions is not clear from the existing evidence.

There is a body of evidence examining factors influencing the prescribing behaviours of doctors, such as lack of guideline knowledge, time constraints, risk aversion, patient pressure, and social norms [14–16]. However, there is more limited evidence of specific barriers and enablers to the implementation of AMS programmes in the hospital setting [17, 18]. To our knowledge, no previous studies have systematically investigated multi-professional factors associated with implementing a nationally agreed AMS programme within the acute hospital setting, across multiple health regions.

The use of theoretical frameworks from the social sciences can aid exploration of implementation challenges in complex situations. For instance, Normalisation Process Theory (NPT) [19–21] has been used extensively to evaluate implementation processes and to aid interpretation and explanation of barriers and facilitators within health care research [22]. The core constructs of NPT relate to the properties of the intervention (*capability* of working and being integrated in practice) and the contribution practitioners make via the mechanisms of *coherence, cognitive participation, collective action, and reflexive monitoring* [23] (explained below). NPT also acknowledges the influence of the *context* of practice, whereby “organisational setting, complex intra- and inter-professional interactions, multiple competing tasks operationalised under pressure” come into play [24] p.291. These factors impact on the individual and teams’ ability to introduce and sustain new practices.

The aim of this paper is to explain the mechanisms influencing implementation of a national programme for AMS in acute-care hospitals across Scotland, using NPT as an interpretive framework to explore multi-professional perspectives. By applying NPT, we address the questions ‘which group of actors’ (e.g. AMTs, doctors, nurses, clinical pharmacists) ‘have which problems’, and ‘what sort of problems are these’?

## Methods

This exploratory qualitative study used in-depth telephone interviews with AMS implementers (AMT lead infection specialist consultants, AMT pharmacists, AMT nurses) from 14 out of a possible 15 regional Health Boards (one board did not have an AMT), and 15 local focus groups with front-line hospital practitioners (prescribing doctors, ward based nurses, and clinical pharmacists) from five purposively selected Health Boards. Criteria for selection of Health Boards as focus group sites was based on documented variation in specific antibiotic use (high or low volume) recorded in the national Hospitals Medicines Utilisation Database (which collects information from hospital pharmacy systems and presents standardised information on medicines usage).

Access for the study was provided by the Research & Development department in each participating Health Board. Approval for the study was granted by the Glasgow Caledonian University School of Health & Life Sciences Ethics Committee (HLS/NCH/17/020). The SAPG Programme Lead acted as a gatekeeper to AMTs in each Health Board, circulating study information to all AMT members, who then contacted the research team to arrange participation. AMT members in the Health Boards that were selected for focus group participation circulated study information to potential participants via posters, email, group meetings, or individual contact; those practitioners who were available and interested then attended planned focus groups at each health board site. All participants were provided with study information prior to voluntary participation and each confirmed written consent.

Interview and focus group topic guides were developed, with broad question areas related to participant’s role or experience related to AMS, perceptions of barriers and enablers to AMS, recommendations to strengthen AMS (Additional file 1). These topic guides were used flexibly to enable in-depth probing of participant responses. Interviews and focus groups were conducted April–July 2018, by experienced researchers (KC, RL, VN), not previously known to participants, and were digitally recorded and fully transcribed before being imported into NVivo 11©software for data management. Interviews and focus groups lasted between 24 and 72 min, averaging 50 min.

May and colleagues [20] indicate the various ways in which NPT can be used to analyse and interpret data; we adopted the recommendation to conduct a standard inductive thematic analysis then structured our interpretation by reframing the themes around NPT constructs, addressing the NPT questions outlined above. Data was analyzed in three stages: firstly, transcripts were inductively coded using Braun & Clark’s [25] principles of thematic analysis; secondly, a content analysis approach [26] was used to extract, condense and categorise (according to already identified themes) a list of factors which acted as barriers or enablers to AMS; finally, NPT was used to explain the properties of the intervention and mechanisms of action which help or hinder the routinisation of AMS into everyday clinical practice. The research was funded as part of a programme of work intended to inform policy; none of the researchers had prior bias or assumptions related to the research topic. Rigour in data analysis was achieved by maintaining an audit trail of coding via NVivo© and peer review of all coding and category development by at least two researchers (KC, RL, VN), with agreement in analytical decisions reached during team discussions. COREQ reporting criteria for qualitative research were applied [27].

## Results

Participant numbers per occupational category are shown in Table 1. All Scottish AMT pharmacists participated (n = 15) and nine out of a possible 14 AMT medical leads were interviewed (five were unable to participate due to workload pressures). All three AMT nurses in-post were interviewed. Front-line practitioners from each included profession participated in discipline specific focus groups at each of the five purposively selected Health Boards i.e. three focus groups per Health Board, five groups per discipline (3–9 per group, n = 72). A total of 99 individuals participated overall.

**Table 1:** Participant numbers per occupational category

Occupational category	Number
AMT Lead Infection Specialist Consultant	9
AMT Pharmacist (1 per 13 boards, 2 from one large board)	15
AMT Nurses (few boards have AMS nurses)	3
**Total AMT participants across 14 NHS Health Boards**	**27**
Prescribing doctors × 5 focus groups (3–6 participants per focus group)	21
Clinical pharmacists × 5 focus groups (3–9 participants per focus group)	28
Ward based nurses × 5 focus groups (4–6 participants per focus group)	23
**Total front-line practitioner participants across 5 NHS Health Boards**	**72**
**Total study participants**	**99**

Thematic analysis of the full data set identified six key themes influential in the implementation of AMS: ‘people matter’; ‘context, time and resources matter’; ‘knowledge experience and confidence matters’; ‘prioritisation matters’; ‘technology matters’; and ‘feedback matters’. Detailed categorisation of all barriers and enablers is available in Additional file 2.

### Operationalising NPT within the study

The following sections apply the lens of NPT to explain the properties of the intervention (capability), contextual elements, and mechanisms of action affecting the implementation of AMS in hospital settings. Participant quotes are referenced by interview or focus group number and professional category (e.g. Interview1 Doctor). Table 2 below maps each theme to the respective NPT component, with a brief explanation of the construct provided at the beginning of each result section.

**Table 2:** Mapping of themes to relevant NPT constructs

***Theme***	NPT Constructs
***People matter:*** (leadership, relationships, staff buy-in, staff continuity)	• Capability / workability • Context • Coherence • Cognitive participation • Collective action
***Knowledge, experience & confidence matters:*** (awareness, education, knowledge, experience; especially for junior doctors & nurses)	• Coherence • Cognitive participation • Collective action
***Prioritisation matters:*** (relative prioritisation given to AMS in relation to competing objectives)	• Cognitive participation
***Context, time and resources matter:*** (size and complexity of the organisation, availability of staff, time)	• Capability / workability • Context • Collective action
***Technology matters:*** (methods of accessing information & communicating; presence or absence of meaningful data)	• Collective action
***Feedback matters:*** (the nature and timing of feedback on audit data)	• Reflexive monitoring

### Capability: how ‘workable’ is the AMS programme?

The multi-level Scottish AMS programme is described in ScotMARAP which outlines the responsibilities of various groups, including SAPG, Health Boards, the AMTs, and antibiotic prescribers [11]. The end-goal of the programme is compliance with SAPG evidence based AMS guidelines. In general, medical staff are familiar with a guideline based approach to prescribing, although a degree of professional autonomy in decision making is normally accepted.

The SAPG AMS guidelines [28] begin by recommending which type, dose, and mode of administration of antibiotic is indicated; this action would normally be undertaken at the point of initial patient assessment and management by the attending doctor. This aspect of the guideline is inherently ‘workable’, as prescribing treatment is an essential part of normal care and any change in practice required relates mainly to the specific antibiotic to be used. However, subsequent components of the guidelines (i.e. recording indication for antibiotic, recommended review of antibiotic at 72 h, switch from intravenous to oral antibiotic, limited duration of antibiotic) all require additional action to be taken, sometimes at a later stage, potentially by different practitioners from the original prescriber and involving more senior staff who influence decision making. Thus, components of the theme *‘people matter’*, may challenge the ‘workability’ of some aspects of the guidelines.

### Context

‘Context’ refers to the ways in which resource and task allocation, social roles, and team norms in the areas in which AMS is to be integrated are negotiated [21]. Of particular interest here is the level of discretion which those involved in AMS implementation have over resources (normative restructuring) and their own and others’ actions (relational restructuring).

Our analysis indicated that a major challenge to the implementation of the AMS programme relates to available resource, which impacts on many of the mechanisms detailed below. This begins with the staff resource available to the AMT, which influences all subsequent implementation actions. In NPT terms, opportunities for normative restructuring, or modifying resource allocation, are constrained; thematically, *‘context, time and resources matter*’. Similarly, hierarchical relationships within teams (Doctors Vs Consultants, Nurses Vs Doctors) may impact on prescribing decision making, with collective action to implement AMS being affected. Opportunities for relational restructuring, i.e. reframing roles and responsibilities, may be limited; *‘people matter’* here.

### Coherence: how do participants make sense of AMS?

The construct of coherence encapsulates the work individuals and groups do to make sense of a new practice, in this case understanding the principles of the AMS programme. It relates to the ways individuals and teams understand the purpose of AMS, recognise the differences between this new way of working and previous practice, understand what is required of them, and thereby construct the potential value of AMS to their work. The themes *‘people matter’ and ‘knowledge, experience and confidence matters’* were influential here.

Coherence was enabled by the strategic work of the national SAPG network, carried forward in each Health Board by the AMT. SAPG brings together a multi-disciplinary group of key stakeholders with an interest in implementing AMS, including representation from all Scottish Health Boards. AMT interviews highlight the value of SAPG in co-ordinating national efforts; enabling networking and sharing of experiences and solutions; generating consensus; and providing an authoritative voice to support local implementation:

“I find it very helpful. I mean, it’s good especially when you’re one person leading the AMT here and trying to further this agenda, to have a national body behind you saying, look, this is what everyone in Scotland is doing, this is what’s appropriate, this is what everyone else thinks is correct, and if you want to have a variance from it then we need to have a pretty good reason behind it. It’s really useful to have that authority behind you.” [Interview4, Doctor]

AMT participants were clear that the national approach afforded by SAPG enabled a collective sense of purpose and value for AMS work. However, translation of this potential into everyday clinical practice requires that front-line practitioners involved in prescribing and administering antimicrobials are also aware of, and can see the value of, AMS.

Raising awareness of AMS is a key function of AMTs. This was enabled across all Health Boards during the induction of new junior doctors and by the availability of local prescribing guidelines. In general, AMT members believed that junior doctors readily accessed local prescribing guidelines and were diligent in using these:

“The willingness of our junior doctors to come on board with things has been really useful … they tend to listen to what is local guidance and policy and they will follow our guidelines really well. So I’ve been quite impressed with them.” [Interview19 Doctor]

However, raising awareness of AMS policies with more established medical staff, or those who were transient, such as locum consultants, was more problematic:

“we don’t really have good conversations with the consultants. … The junior doctors tend to follow guidelines very well. However, we know that they defer to their seniors for decisions about antibiotic treatment. So if we’re not having much linkage with registrars, specialist registrars and consultants to update them in stewardship messages and the reasons why…” [Interview5 Pharmacist]

Similarly, whilst some AMTs were engaged in targeted AMS training activities with nurses, education for nursing staff was generally recognised as being insufficient.

“I think enabling nurses to be released from the wards is challenging. The staffing levels are perhaps so low, the acuity within the wards is so high, that nurses probably aren’t getting the chance to come out for 30 minutes to an hour from clinical time.” [Interview10 Nurse]

This view was supported by many nursing focus group participants. Whilst some nurses reported mention of antibiotic use during intravenous therapy training, and some were aware of the NHS Education for Scotland (NES) AMS workbook for nurses, most reported limited awareness of stewardship or the nurse’s role therein.

“I think because it's *(AMS)* something that has never been discussed as part of my role, I've never ever thought well...questioned myself about it” [FG4 Nurses]

Some examples of excellent work to raise awareness and engage with nurses around AMS were provided, however these were generally a result of targeted projects in a limited number of wards, and were not commonly part of AMT activity in most Boards. Thus, for many, lack of time and resource to engage with nursing staff created a significant barrier to raising awareness of AMS for nurses and challenged their ability to make sense of the issues.

Findings indicated that SAPG and AMTs have clarity around the purpose, value, and demands of AMS. In general, junior doctors in all Boards have well-established opportunities to understand the requirements of AMS via induction training and the accessibility of guidelines – AMS has coherence for them. Conversely, problems of coherence lie primarily in relation to limited accessibility or uptake of AMS information for other practitioner groups, across all Boards.

### Cognitive participation: how is ‘buy-in’ to AMS achieved?

The construct of cognitive participation relates to the work that goes on at various structural levels to secure the consent, co-operation and ongoing support of other players. The themes *‘people matter’* and *‘prioritisation matters’* are influential in this construct; despite energetic leadership by AMTs, competing priorities may serve to undermine support for AMS.

Effective leadership by AMT lead consultants and AMT pharmacists is fundamental to the initiation and legitimisation of AMS. Engaging with and directly influencing others seems core to their success.

“I think my predecessor is the reason why it *(AMS)* works so well…. she is able to make sure that people who would never come to AMT, like an orthopaedic surgeon, comes every single time and doesn’t feel like he’s being dragged there kicking and screaming.” [Interview19 Doctor]

Conversely, lack of leadership influence was noted to inhibit AMT effectiveness, with restrictions on commitment or capacity mentioned by some:

“there's been a bit more kind of difficulty, I suppose, in getting someone to lead the AMT. I think people have been more sort of cajoled into doing it sometimes … that's been a bit of a stumbling block” [Interview14 Pharmacist]

In addition, the importance of buy-in and clinical leadership from medical consultants was highlighted; AMT participants particularly noted the influential role that senior medical consultants have on junior doctors:

“…and if they’re *(consultants)* enthusiastic, then that trickles down through the rest of the team. So if they see the consultant going to the guidelines and seeing what it is they should have patients on … they then realise that that’s what’s expected of them.” [Interview23 Pharmacist]

However, most AMT participants noted variation in the commitment and buy-in from different medical specialties, with some evidence of a general pattern across different regional boards:

“I think it’s very variable, part of that relates to specialty. So we’ve got a bigger buy-in from the medical consultants than we do from the surgical. Now, we have buy-in from the orthopaedics, but the general surgeons, they are… we’ve struggled to get engagement with them. And because we’ve not got a buy-in from the consultants, we’ve not got the buy-in from their junior staff.” [Interview21 Doctor]

Opportunities for nurses to engage in AMS were limited in most boards. A variety of reasons were offered by participants for lack of nurse enrolment in AMS, ranging from nurses *‘lacking confidence’,* feeling disempowered, to nurse managers seeing AMS as the work of prescribing doctors and not a task they wanted staff to take on as a nursing role.

“when we’ve tried to engage nurses in IVOST *(switching from intravenous to oral antibiotics),* sometimes it’s worked, but … when I’ve asked them why *(they don’t)*, they said that they don’t feel empowered to do it. They say the doctors don’t listen to them.” [Interview1 Pharmacist]

“Any conversations I’ve had with them *(nurse managers)*, some are more supportive than others; but, to be honest, I think they’re just so ‘trying to man the wards’, they’re just so kind of ‘let’s just keep our head above water’ that anything in addition to that is a sort of an extra ask almost.” [Interview8 Pharmacist]

For AMT participants, AMS was a clear priority, however, not all stakeholders attributed the same importance to AMS. In some medical specialities, the needs of their patient group were viewed as ‘different’ and obtaining buy-in to AMS guidelines was challenging:

“… haematologists, I think in that area there is a practice that everybody must get everything *(antibiotics)* because there’s so much to lose, and that’s coloured by experience…. I hear haematologists talk about people developing septic complications of chemotherapy and dying rapidly with multi-drug resistant bugs and then that is translated into, well, we have to treat everybody who has an infection with this sort of blunderbuss therapy.” [Interview2 Doctor]

For others, the multitude of competing priorities generated a barrier to prioritising AMS:

**“**We compete with lots of different initiatives at lots of different times, so for example, it’s the national sepsis day or something, and sepsis *is* very important … but, our experience is that the management of sepsis tends to mean that more people get given antibiotics almost on a ‘just in case basis’ … so it’s an issue between us, that that *(sepsis six)* almost promotes additional antibiotic use when we’re trying to stop inappropriate antibiotic use” [Interview4 Pharmacist]

The work of cognitive participation was neatly summed up by this participant:

“I think the biggest thing is making it part of everyone’s job, everyone’s life, as opposed to just being designated to the chosen few that happen to have it as their job …. I think that’s the biggest message we need to sell is that everyone has a role to play in stewardship and it’s not just the antibiotic pharmacist and the micro guys.” [Interview23 Pharmacist]

Overall, whilst AMTs and most junior doctors were committed to the principles of AMS, some challenges remain in selling the importance of the AMS message to specific groups of medical consultants. Similarly, resource constraints or role definitions limited efforts to enlist nurses in AMS. The ‘sepsis six’ care bundle initiative also presented a barrier to the prioritisation of appropriate prescribing messages. However, where there is effective and energetic AMT leadership engaging widely with relevant stakeholders, endorsed by other medical consultants and opinion leaders, participants readily agree that AMS should be part of their work and support the use of AMS guidelines.

### Collective action: what do people do to enact AMS guidelines?

Collective action refers to work undertaken by individuals and groups, at different structural levels, to enact the practices associated with AMS. It requires the organisational context to be appropriately supportive; for individuals to have the necessary skills and to perform the tasks associated with AMS; and for different stakeholders to trust in the AMS related work carried out by others. Fundamentally, this construct answers the question ‘how does the work of AMS get done?’ Once again, the theme *‘people matter’* is important, here the barriers presented by lack of medical staff continuity is prominent. *‘Knowledge, experience & confidence’* is also influential, often with the interpersonal dimension of having confidence to apply knowledge in team-based interactions being key. Hierarchical relationships within medical teams often impacts on the prescribing behaviours of more junior doctors.

The ability of these factors to impede effective AMS actions are illustrated in the quote below.

“so you get told on a ward round to start these things and then you look at their swab samples then you think, that doesn’t make any sense from what we’re told in our app *(AMS guidelines)*. So you then phone micro and micro say, ‘no, no, no, do that’ and you change it, and the consultant gets annoyed the next day or you’re not there on the ward round because you’ve gone off shift and you go back a week later and they’ve been put back on the old antibiotics.” [FG2 Doctors]

Nurse participation in AMS varied, and whilst there were examples of nurses working in specialist roles being actively involved in stewardship conversations, a more typical description of the limited involvement of nurses in stewardship is offered below:

“I’m just at ward level giving out the antibiotics that are prescribed.” [FG14 nurses]

Nurses’ confidence to challenge doctors in potentially hierarchical relationships also varied:

“It depends on what experience you’ve got. There are some doctors who do not take kindly to being reminded of those things. Whereas others are very approachable and will listen to you. Some will ignore you just to prove a point.” [FG14 Nurses]

Challenges in *‘context, time and resources’* often create significant barriers to collective action, primarily via restrictions on staff resource and therefore time available for the different component activities of AMS. Whilst these challenges affect all staff groups, the capacity for clinical pharmacists to engage in AMS at ward level varied markedly across Health Boards: some incorporate AMS activity in their normal work, including reviewing all prescription charts and discussing issues related to antimicrobial prescriptions with clinical staff; others visit wards only to manage discharge prescriptions and have very little involvement in routine management of other prescriptions:

“Well, from the moment a patient is admitted, they're looking for their discharge moment, not day, their moment. And that takes priority over everything else. You can be quite involved in really quite serious clinical work, but you will have flow coordinators, and other such people, looking over your shoulder telling you, you should be doing the prescription because they want that patient out. That’s all they care about.” [FG8 Pharmacists]

The physical environment was also influential, and related to this, ‘technology’, or lack thereof, can either support or hamper AMS work, as shown below:

“there are a number of issues. One is time, so now we’ve moved to our new hospital, their ward rounds are taking longer, physically taking longer to do …part of that relates to the layout of the wards and part of it relates to IT issues, so we’ve gone to a hospital that’s all single rooms and they’re having to log into computers in each single room. We have an electronic prescribing system, so they’re having to log in each time to review the electronic prescribing system, to review antibiotics [*that takes additional time]* so they essentially are not doing that in a timely way on the ward rounds.” [Interview21 Doctor)

Therefore, a variety of factors may enable, but often constrain, the ability of different practitioners to collectively work towards delivering the AMS programme.

### Reflexive monitoring: what do people do to appraise the consequences of their contribution to AMS?

This construct relates to the work participants are involved in when trying to understand and evaluate a complex intervention such as AMS in practice. By monitoring practice, areas for improvement become apparent and further support can be offered. This relates directly to the theme *‘feedback matters’.* Whilst AMTs in all Health Boards undertook monthly audits of prescribing indicators, reported to SAPG and collated nationally, there is an evident dichotomy in activities thereafter. Some AMTs ensured direct, point-of-care feedback to practitioners, for example

“when we go to the ward to collect the data we would always let the staff know, the medics and the nursing staff, that we have arrived on the ward and we’re doing this audit; and then once we’ve finished the audit, we’ll give the senior charge nurse a verbal feedback as to how the ward has done, whether that be good or bad, and we would also let the medics know this is what we’ve found and you’re either doing great, carry on, or we’ve found a couple of things a wee bit wrong, can you just ….” [Interview9 Pharmacist]

Others do not provide any direct feedback and therefore practitioners are not aware of their AMS performance compliance and have little opportunity to improve prescribing quality:

“so we have collected a huge amount of data over the last wee while through (audit of) prescribing indicators. But I don’t think we’d used it, so we collected the data … but we hadn’t shared it with the clinical teams that were from where we had collected it.” [Interview12 Physician]

“I've never had any feedback. I am aware that the audit is happening, but I've never had any feedback.” [FG3 Doctors]

Similarly, whilst a few AMTs use audit data to target quality improvement projects, most report no capacity for quality improvement work and therefore opportunities for the reflexive component of monitoring are lost.

“I have to say I don’t think all the audit programme is necessarily as useful as it could be. I think we collect a lot of data and we don’t have the capacity to do the QI improvement work that should be done alongside it; that’s my bugbear about it.” [Interview7 Pharmacist]

Our findings suggested significant variation in the level of passive or active feedback of prescribing data to front line prescribers, with some exemplary practice contrasted with resource constraints limiting direct feedback and quality improvement opportunities.

### Summary of findings

The previous analysis detailed the properties/workability of the AMS intervention and the mechanisms of action across each specific NPT construct. Using NPT can then reframe interpretation of data to address the questions ‘*which group of actors have which problems*, and *what sort of problems are these*? Based on the preceding analysis of activity within each NPT construct, Table 3 provides a summary response to those questions.

**Table 3:** Summary of analysis

Actors	Capability	Context	Coherence	Cognitive participation	Collective action	Reflexive monitoring
AMT		Limitations on organisational support to resource / prioritise AMT work. Limited availability of technical solutions to support prescribing review.		Constraints on AMT leadership engaging with all stakeholder groups.		Lack of provision of direct feedback of indicator audits to clinicians.
Prescribing doctors	Lack of continuity in medical cover makes ongoing review of prescribing decisions challenging.	Medical hierarchies create limited ability to influence team norms or practices.			Lack of confidence to challenge consultant decisions.	No feedback on prescribing indicator audits, therefore no reflection on personal practice.
Consultants or locum medical staff			Lack of provision of or engagement with AMS updates.	Competing issues impede prioritisation of AMS.	Lack of continuity of medical staff impedes ongoing AMS activity.	Limited feedback on prescribing indicator audits, therefore no reflection on personal practice.
Nurses		AMS often not viewed as a nursing role or responsibility. Limited opportunities for engagement.	Lack of time and access to AMS training.	Lack of awareness of potential nurse’s role in AMS.	Lack of engagement in AMS activities. Lack of confidence to question doctors’ decisions.	
Clinical Pharmacists		Resource constraints and role priorities which limit opportunities for AMS related activities.				

## Discussion

To our knowledge, this is the first study to investigate the implementation of a national AMS programme from a multi-level, multi-professional perspective, using NPT to explain challenges in the process of implementation. A further strength is the sampling strategy, enabling inclusion of AMT implementers from all Scottish Health Boards and groups of front-line doctors, nurses and clinical pharmacists from five diverse Health Boards. The relatively large sample size (n = 99) for a qualitative study, the diverse geographical distribution of participants, and the multi-professional nature of the data gathered means we are confident that sufficient data with ‘information power’ was generated to reflect the nature of the phenomenon [29]. We believe these findings are transferable to similar health care contexts internationally, although we make no claims regarding generalisability of the results. In particular, we recognise the potential limitation of self-selection of focus group participants, albeit their employing health boards were purposively sampled.

In considering the work done to implement AMS, this discussion focuses on the multi-professional groups of ‘actors’ involved and the typical challenges they faced. The role of SAPG nationally and AMTs at Health Board level provided an implementation approach with significant potential to impact on prescribing behaviours by engaging, informing, advising, monitoring, and providing feedback to front-line practitioners. This study identified many examples of excellent AMT practice in all these areas, however, variation in the resource allocation, leadership capability, and the nature of the specific activities undertaken by AMTs in each Health Board means that there remains significant scope for AMT potential to be further realised. As discussed by Huttner and colleagues [30] in a narrative review of AMS success stories from around the world, sharing obstacles and success stories in implementing AMS programmes enables the global stewardship community to benefit from the experience of others. Our study provides one such exemplar, using NPT to explain those mechanisms which help and hindered implementation in the Scottish acute care context.

Our evidence suggests that for most medical practitioners, AMS guidelines have inherent workability and coherence: in NPT terms, they are differentiated from previous antibiotic prescribing practice; individuals and teams agree the purpose of AMS; the availability of guidelines means that participants understand what is required of them; and the approaches to AMS are valued as part of prescribing work. However, working patterns often led to a lack of continuity in medical staff involvement in patient care. This could fracture the workability of AMS beyond initial prescribing, with key aspects ‘falling between the gaps’, particularly in relation to review of antibiotic use. This difficulty has been recognised elsewhere and additional mechanisms to trigger review have been advocated [31, 32].

Particular tensions were found in our study between AMS principles and heightened concerns around sepsis. Broom and colleagues [33] also found that perceived immediate clinical risks for individuals tend to dominate doctor’s prescribing decisions over longer term population health risk. Greater challenges were reported in engaging some consultant level doctors, particularly in surgical, respiratory and haematology where AMS may not be prioritised due to perceived specialist patient needs. Consultant preferences, which may conflict with AMS guidelines, were found to be influential on junior doctor prescribing actions. Likewise, Broom and colleagues [16] who argued for a sociological understanding of antibiotic misuse in the hospital sector, found doctors’ experiences of interpersonal and intra-professional pressures, and the role of localised norms, influenced prescribing behaviours. The potential contextual barrier of medical hierarchies and role expectations was also described by Charani and associates [34], who highlighted the role of ‘prescribing etiquette’ in determining the cultural rules within medical prescribing practice. As consultants present a powerful social influence on the behaviour of doctors in training, their engagement in AMS is an area for ongoing attention.

With some isolated exceptions, we found the contribution of nurses in promoting AMS was under-utilised, with dominant role expectations and resource constraints presenting contextual barriers to participation. Limited opportunities for training meant that nurses were often not aware of their potential contribution to AMS; for nurses, the AMS process lacked coherence therefore potential cognitive participation and collective action were severely constrained. There is growing discussion in the literature advocating greater nurse involvement in AMS [35–37], with other exploratory studies finding comparable results to ours [38–40]; however, intervention studies which detail approaches to strengthen the nurse’s role are limited [41, 42]. As nurses are the most consistently present members of the healthcare workforce, capitalising on the continuity afforded by nurses to prompt prescribers, who are more likely to be transient, would be of significant benefit in strengthening AMS.

Whilst the specialist AMT pharmacist was acknowledged as fundamental to the success of AMS programmes, the capacity for other clinical pharmacists to engage in AMS at ward level varied markedly across Health Boards. The idea of AMS was coherent for them and participants reported wishing to be more involved, however they were largely impeded in collective action by challenging contextual factors such as resource allocation for the various components of their role, with an emphasis on discharge planning to the detriment of other elements. Time constraints were also reported by Weier and colleagues [43], in a cross-sectional survey of Australian and French hospital pharmacists (*n* = 259). Similarly, the need for protected time for stewardship within a supportive organisational culture was highlighted in a small qualitative study of pharmacists in the USA’s Veterans Affairs centres [44]. The potential for the wider clinical pharmacist workforce to engage more directly with prescribers than is possible for the specialist AMT pharmacist may be worthy of further consideration.

The benefits and challenges of providing effective feedback on prescribing indicator audit to enable reflexive monitoring were highlighted across participant groups. The potential impact of audit and feedback in changing professional behaviour is well-recognised, with the conclusions from Ivers and colleagues’ 2012 Cochrane review being relevant here [45]. However, resource constraints affected AMTs’ ability to provide active feedback to prescribers in many regional Boards.

Underpinning many of the implementation challenges for individual actors outlined above, organisational context, particularly staff resource and availability of relevant technology to support working practices, were highlighted as exerting an influence on AMS implementation. Attention to these contextual components is required to gain maximum traction in improving AMS further.

## Conclusions

Our study highlighted the beneficial impact of a national approach towards AMS, with strategic leadership rolled out at local level. Findings have illustrated key enablers and important barriers to the implementation of AMS, examined through the lens of NPT.

Future attention should be directed towards the organisational context and resource requirements for AMS implementation. Developing AMT leadership skills, as well as practitioner confidence and knowledge via accessible education, and capitalising on the potential contribution of nurses should be considered. Technological solutions, such as electronic prescribing and other systems which provide behavioural prompts, in addition to feedback mechanisms to reinforce positive change, are recommended.

This study has important implications internationally for others seeking to implement AMS in the hospital setting. There is evidence from international studies [12, 13] that AMS interventions can have beneficial impact on appropriate antibiotic prescribing and reductions in antibiotic resistant infections*.* The challenge for healthcare providers is to maximise the impact of such interventions by focusing on implementation processes which enable the sustainable integration of AMS into routine practice.

## Supplementary information

**Additional file 1.** BEAMS Project: Interview Topic Guide.

**Additional file 2.** Mapping of BARRIERS AND ENABLERS – Individual interviews & Focus Groups.

## Data Availability

The qualitative transcript datasets generated and/or analysed during the current study are not publicly available due to risk of identification of individuals or organisations, but are available from the corresponding author on reasonable request.

## Abbreviations

AMR: Antimicrobial resistance
AMS: Antimicrobial stewardship
AMT: Antimicrobial Management Team
NPT: Normalisation Process Theory
SAPG: Scottish Antimicrobial Prescribing Group
