# Supplementary material for: Mechanisms affecting the implementation of a national antimicrobial stewardship programme; multi-professional perspectives explained using normalisation process theory
Source: Antimicrob Resist Infect Control. 2020 Jul 2;9:99. doi: 10.1186/s13756-020-00767-w (PMC7330968; doi:10.1186/s13756-020-00767-w)
Supplement: Supplementary file 1 — Additional file 1. BEAMS Project: Interview Topic Guide. [file 13756_2020_767_MOESM1_ESM.docx]

**BEAMS Project: AMT individual telephone interviews**

**The interview**

1. Firstly, can you tell me about the antimicrobial stewardship programme in your area?
2. From your experience, what factors might present barriers to antimicrobial stewardship?
3. Within your board, are there any factors that you think have enabled the implementation of antimicrobial stewardship programmes?
4. To what extent do you think colleagues / other people in your Board support antimicrobial stewardship programmes? What are the key roles or functions in AMS, beyond the Antimicrobial Management Team?
5. Are staff given information about their area’s performance regarding AMS or AB prescribing - who / how/ when? How do you think staff respond to feedback (if at all)?
6. What do you think might be done to strengthen antimicrobial stewardship programmes in your area / nationally?
7. What would help you in your role in AMS?

**Finishing the interview** Thanks for your time. Have you any questions, further points to highlight?

**BEAMS Project: Staff focus groups**

1. Firstly, can you tell me about antimicrobial stewardship in relation to your role?
2. From your experience, what factors might present barriers to your role in antimicrobial stewardship? Why might you not apply the guidelines?
3. Within your area, are there any factors that you think have supported your role in antimicrobial stewardship?
4. To what extent do you think colleagues / other people in your Board support the antimicrobial stewardship programme?
5. Are staff given information about their area’s performance regarding AMS or AB prescribing - who / how/ when / is (or would it be) helpful?
6. What do you think might be done to strengthen antimicrobial stewardship in your area / nationally?
7. What would help you in your role in AMS?

**Finishing the discussion** Thanks for your time. Have you any questions, further points to highlight?
