# Supplementary material for: Mechanisms affecting the implementation of a national antimicrobial stewardship programme; multi-professional perspectives explained using normalisation process theory
Source: Antimicrob Resist Infect Control. 2020 Jul 2;9:99. doi: 10.1186/s13756-020-00767-w (PMC7330968; doi:10.1186/s13756-020-00767-w)
Supplement: Supplementary file 2 — Additional file 2. Mapping of BARRIERS AND ENABLERS – Individual interviews & Focus Groups. [file 13756_2020_767_MOESM2_ESM.docx]

**BARRIERS AND ENABLERS – Individual interviews & Focus Groups**

| **BARRIERS** | **ENABLERS** |
| --- | --- |
| - **‘People matter’** (leadership, relationships, buy-in, continuity, ability to interact at personal level) | |
| Lack of AMT Leadership  Lack of AMT engagement from certain groups of medics, senior clinicians  Lack of senior management engagement  Lack of engagement with specific groups: nurses  Lack of nursing involvement  Lack of communication (including documentation) between departments, between prescribers and those administering and between senior consultants and juniors  Lack of ward rounds/MDT meetings (lack of face-to-face engagement | Senior support, AMT engagement and leadership  Good relationships/ working with enthusiastic, interested people  Engagement activities: ward rounds, Face to face interaction  Engagement and links with specific people e.g. IPC team, microbiology, ID, clinical pharmacists, medical staff and specialities, nurses  Good relationships/team working  Good engagement with pharmacists  Good engagement with nurses  Active multi-disciplinary AMT  Ward rounds/patient reviews/presence on the ward (all face to face measures) |
| - **‘Environmental context, time and resources matter’; at all levels** | |
| Lack of time and resources: AMT resource, AM nurse, time for nurses to attend education, pharmacist time, microbiology resource, staff shortages  Lack of junior doctor continuity  Lack of clinical pharmacist engagement – no time for AMS  Organisational Structure – challenges of multiple sites, single rooms, large organisation, small organisation, not enough IPC link, geography, lack of coordination and communication i.e. difficult to disseminate guidance  Resource issues –people and time  Lack of doctor continuity/working patterns | Smaller health board, more day-to-day access to clinical colleagues  Drug shortages/restricted prescribing |
| - **‘Knowledge, experience & confidence matters’** (awareness, education, knowledge, experience; esp for Junior Drs & nurses); | |
| Lack of knowledge, experience, confidence, lack of clear evidence  Lack of engagement with specific groups: nurses  Lack of knowledge/confidence/awareness  Individual prescribing/ not adhering to guidelines (senior staff, surgical specialities) | Junior Dr induction  Education/teaching e.g. short presentations on the ward, involve nurses  Innovative education/engagement strategies (on the ward) |
| - **‘Technology matters’** (methods of accessing information & communicating; presence or absence of meaningful data) | |
| IT/technology issues -signal coverage, app issues, intranet, HEPMA, no electronic prescribing, no prompts, lack of technology systems and do not link up with national systems  IT issues: difficulties with HEPMA, intranet, no electronic prescribing  Guidelines not updated/completed  Poor documentation or lack of documentation  Practice issues e.g. lack of review, missed doses, misuse of AB alert forms, drug shortages, more complex patients | Accessible, updated guidelines  IT/technology e.g. Electronic prescribing, Electronic alerts, Electronic guidance  Good IT systems e.g. app, triage  National surveillance programme  Accessible, easy-to-follow, up-to-date guidelines  Good documentation/prompt sheets/stickers |
| - **‘Feedback matters’** | |
| Lack of audit feedback to clinical teams  Lack of feedback/audit | Audit and feedback -real time point of care feedback to prescribers |
| - **‘Prioritisation matters’** | |
| Lack of commitment to AMS outside of core AMT  Competing priorities / Not seen as priority  Beliefs regarding AMR (patient need versus greater good), tension between AMR and sepsis  Competing priorities  Sepsis six | External support from ASAP, SAPG, AM nurses group |
